# Dr. Dalton's Vivisections

**Published:** 1854-03

**Authors:** 


*Dr. Dalton's Vivisections.* — We owe an apology to our readers that we have not, in a preceding number of the Journal, informed them of these most interesting experiments. During the past few weeks, Dr. Dalton has, in illustration of his course of lectures upon Physiology at the Buffalo Medical College, performed a great number of experiments upon the living animal, illustrative of the natural functions. Many of the mooted points, as well as the received doctrines of physiology, have been subjected to the test of vivisection.

Our engagements have prevented us from witnessing so many of these as we should have been glad to do. Among the most interesting of those at which we have been present, was a series upon the function of the larynx, and the pneumogastric nerve.

Dogs were selected for vivisection. A middling-sized black slut was brought in under the influence of ether, the operation of exposing the larynx having been already performed. Upon everting the larynx so as to bring into view the chink of the glottis, the respiratory movements, which became more complete as the animal recovered from the influence of the anæsthetic, were seen to consist of a separation of the cricoid cartilages, and a consequent dilatation of the opening, synchronous with the act of inspiration, and a narrowing, but not entire, closure of it during expiration. As the animal recovered, and its cries became vigorous, the action of the cartilages was more distinct, though not as much so as in a previous experiment upon a larger dog, at which Prof. Agassiz and others were present.

The recurrent laryngeal nerve of one side was now severed, with the result of paralysis to the intrinsic laryngeal muscles of that side. On cutting the nerve of the opposite side, this paralysis became complete. The respiratory function was still perfect, except so far as the larynx was concerned. The cricoid cartilages fell together at the inspiration, impeding greatly the passage of air to the lungs, while the expiratory movement was attended by an opening of the fissure from the propulsive force of the air behind. The function of these nerves was now evident. They controlled the movements of the larynx, and by the contraction or relaxation of the vocal chords, they intensify and modulate the tone and pitch of vocal sounds.

The pneumogastric was now divided upon each side. The paralysis was then more complete; the inspiratory movement was labored, while the expiration was at once prolonged and forcible.

The animal was now killed by severing the spinal cord at its junction with the medulla oblongata.

A puppy, six weeks old, was now laid upon the table. The laryngeal nerves were severed, and the same result attended the respiratory movements; but it was evident that all the difficulty of breathing proceeded from the larynx, and that the function of the lung, as well as the sensations of the animal, were unimpaired.

The pneumogastrics were now divided. With greatly increased difficulty of respiration, there was no apparent increase in the distress of the animal.

The muscular tissues of the bronchial tubes were paralyzed; communication between the brain and lungs was cut off; the animal, though incapable of perfectly performing respiration, was at the same time unconscious of the wish to respire. The expiration was prolonged, and the inhalations grew gradually less frequent. He was then killed in the same manner as the preceding animal.

Another puppy, of the same age, was now produced, upon which the pneumogastrics had been cut five hours previously. His respirations, previous to the operation, were over thirty per minute. They were now about five per minute. The animal, though not unconscious, was indifferent to external impressions, and lay quietly upon the table, raising his head slowly with each inspiration. He might live some hours yet. Upon some very young dogs it had been found that division of the pneumogastric was followed by immediate death; but an older animal would sometimes live five or six days.

Death from division of the pneumogastric was caused, mediately, by imperfect hematosis, owing to the want of the wish to respire, the imperfect entrance of air through the larynx, and the very partial expulsion of old air from the lung. This air became gradually charged with carbonic acid, and the animal died by coma.

This animal died during the night succeeding the lecture, having lived some eighteen hours from the time of dividing the nerves.

On the succeeding morning a small dog was etherized, and his epiglottis excised. On recovering from the anæsthetic, he drank milk without difficulty, none of it finding entrance into the glottis. It is evident, from this experiment, as well as from beholding the close adaptation of the sides of the glottis to each other in the previous experiments, that the function of the epiglottis is not that ordinarily assigned to it. It may partially cover, but cannot close, the larynx. During the act of deglutition, the larynx is drawn upward and forward, the tongue is forced backward, and crowds before it the epiglottis, thus covering in and protecting the laryngeal orifice; but the cricoid cartilages are the efficient organs. It is probable that the epiglottis is rather an elegance than a necessity.

We close this brief and imperfect report by mentioning an experiment performed, on a subsequent day, on a large black slut, relative to the functions of the heart. The thorax was opened, under ether, and the motion of the heart maintained by artificial respiration. The main point of interest in this experiment, lies in the fact that Prof. Dalton has thus verified the phenomenon first announced by his colleague, the Prof. of Anatomy, Dr. E. M.

Moore, some twenty years ago, from his vivisections made at that time. It was found that the heart does elongate during its ventricular systole, having a spiral motion from left to right, which throws up the apex. The fact of elongation, which was sufficiently proved by Prof. Moore, *seems* so improbable, that many physiologists have disputed it.

In an edition of "Bellingham on Diseases of the Heart," just published in Dublin, for a copy of which we are indebted to its author, we find the following upon the ventricular systole:

"During their systole the parietes of the ventricles become pale, hard, and convex; the vertical and transverse diameters are diminished, *the apex is approximated to the base*, and describes a spiral motion from *right to left*, and from behind forward, coming in contact with the parietes of the thorax between the cartilages of the fifth and sixths ribs on the left side, when the impulse of the heart is felt."

Dr. Sibson, another observer, says: "Wherever the junction of the left ventricle to the auricle could be observed, the apex and base of the ventricle were seen *to approach each other steadily during the whole systole.*"

Mr. Bellingham further remarks, that "in the experiments performed by Drs. Pennock and Moore, the apex of the left ventricle was not observed to be approximated to the base during its systole. The expulsion of the blood from the ventricle (they observe) is effected by an approximation of the sides of the heart only, and not by a contraction of the apex toward the base; during the systole, the heart performs a 'spiral movement, and becomes elongated.' All other observers have, however, noticed the shortening from above downward of the ventricular portion of the heart during the systole, and from the manner in which the fleshy columns of the valves are inserted, such would appear to be essential to the perfect action of the auriculo-ventricular valves, when the ventricles contract."

So distinguished an anatomist as M. Cruvielhier, says, that during their systole "the ventricles diminish in all their diameters, the appearance of shortening being most perceptible in the vertical diameters."

Here is an issue between Drs. Bellingham, Sibson, and Cruveilhier, on the one side, and Drs. Pennock, Moore, Dalton, and Lee, upon the other. We have entire confidence in the powers of observation of all these American gentlemen. Dr. Moore's experiments were very numerous, and were confirmed by many intelligent bystanders. Dr. Dalton is so much in the habit of making vivisections that his faculty of appreciating rapid movements is cultivated, and gives his experiment increased value, as we thus gain confidence in his accuracy.

Our *amor patriæ,* as well as our judgment, inclines us to side with the Buffalo Professors. So far as it is a mere matter of observation, their eyes are as reliable as if they had first opened on the other side of the Atlantic. If we look a moment at the theory of Bellingham, we recognize, first, the error of assigning to the heart a motion from right to left during the systole. This may be a misprint, but it should be recollected that Dr. Bellingham is an Irishman, and is, therefore, entitled to his "bull."

It is difficult to explain the elongation of the heart in ventricular systole. The action of the columnæ carneæ would evidently go to shorten the ventricular cavity. The papillary muscles could have little influence, attached, as they are, to the valves, and only serving to prevent their being pushed beyond the point of complete closure.

But the mass of muscular fiber is either spiral, or, as we approach the interior, circular. Necessarily the contraction of these spiral and circular fibers could not shorten the heart. The packing together during systole of spiral fibers overlying the circular, seems to us to explain the elongation. If this would produce it, there are not longitudinal fibers enough to interfere very much in the process. The thing may be illustrated by placing the two hands together at their extremities, but with the palms separated. By bringing them together, the sides of cavity between them are approximated, while the cavity itself is elongated. H.

